# Homogeneous nucleation of corundum nanocrystallites by rapid heating of aluminum formate hydroxide-based precursor powder

**DOI:** 10.1038/s41598-019-51156-2

**Published:** 2019-10-17

**Authors:** Michiyuki Yoshida, Yuta Kato, Yasunori Oumi, Osamu Sakurada, Makoto Tanaka, Masashi Wada, Satoshi Kitaoka

**Affiliations:** 1 Gifu University, Faculty of Engineering, 1-1 Yanagido, Gifu, 501-1193 Japan; 2 Japan Fine Ceramics Center, 2-4-1, Mutsuno, Atsuta-ku, Nagoya, 456-8587 Japan

**Keywords:** Chemistry, Materials science, Nanoscience and technology

## Abstract

The high density nucleation of α-Al_2_O_3_ nanocrystallites was observed by rapid heating of the aluminum formate hydroxide-based precursor powder at 1200 °C for 50 s. The nucleation of α-Al_2_O_3_ nanocrystallites with less 10 nm in size from high purity aluminum oxide matrix has not been observed to our knowledge. Based on the results of XRD and TEM, α-Al_2_O_3_ nanocrystallites nucleated from the amorphous phase which formed after thermal decomposition of the precursor powder. Subsequently, α-Al_2_O_3_ with hollow rod-like morphology formed through coalescence and growth of nanocrystallites after heating at 1200 °C for 1 min. The results obtained in this paper indicates a possible beneficial effect of the rapid heating and cooling of the aluminum formate hydroxide-based precursor powder on the precipitation of α-Al_2_O_3_ nanocrystallites.

## Introduction

Aluminum oxide (Al_2_O_3_) has various structural polymorphs. The most stable phase of α-Al_2_O_3_ (corundum, sapphire) is an extremely important material due to its great hardness, high thermal stability and chemical inertness. These excellent properties and stable mass production techniques have led it to be widely applied in the refractory industry^1^. Moreover, α-Al_2_O_3_ with a purity of 99.99% has been applied to advanced ceramics for many functional applications such as the highly fluorescent oxide ceramics and the fillers to improve the durability of resin. High-purity α-Al_2_O_3_ is synthesized by thermal decomposition of ammonium alum^2,3^ and hydrolysis of aluminum alkoxide^4,5^. In these processes, so-called transition phases (denoted as γ, η, δ, υ) form prior to α-Al_2_O_3_^6–10^. The crystal structures of these phases can be classified by the oxygen sublattice and the interstitial sites for aluminum ion. Metastable phases are based on face-centered cubic packing of Oxygen (fcc) with aluminum ions in tetrahedral and octahedral interstitial sites. α-Al_2_O_3_ has a rhombohedral structure where the oxygen ions form a compact hexagonal sublattice with aluminum ions occupying 2/3 of the octahedral interstitial sites.

It is well known that α-Al_2_O_3_ nucleation within transition phases is sporadic rather than uniform^11,12^. Therefore, the calcination above the temperature of 1200 °C for several hours is necessary to achieve a complete transformation to α-Al_2_O_3_. Higher calcination temperature promotes mass transport, and causes difficulties to obtain fine particles and morphological control. Many efforts have been made to achieve uniform nucleation of α-Al_2_O_3_. The seeding of the precursor is a common route to increase nucleation density. Rajendran^13^, in a study of production of ultrafine α-Al_2_O_3_ powder from aluminum nitrate, found that with α-Al_2_O_3_ seed in the dry gel of aluminum hydroxide, the temperature of the γ-Al_2_O_3_ to α-Al_2_O_3_ phase transformation was lowered to 950 °C. Due to the relatively low α-Al_2_O_3_ formation temperature, the particle size of synthesized α-Al_2_O_3_ powder was 60 nm. But the observed nucleation behavior of α-Al_2_O_3_ was not uniform and still sparse. Sanxu^14^ reported that α-Al_2_O_3_ nanoparticles were uniformly nucleated in α-Fe_2_O_3_ matrix by heating the α-Al_2_O_3_ precursor powder with Fe^3+^/Al^3+^ molar ration of 5 at the temperature of 770 °C. By removing α-Fe_2_O_3_ matrix through selective corrosion, disperse equiaxed α-Al_2_O_3_ nanoparticles with an average sizes below 10 nm and narrow size distributions were obtained. But this separation process is complicated and the experimental operations are difficult. To our knowledge, uniform nucleation of α-Al_2_O_3_ nanocrystallites in aluminum-oxide matrix has not been reported.

Recently, the metal organic precursor of aluminum formate has been studied as a simple and innovative approach to synthesize α-Al_2_O_3_ powders^15,16^. The advantage of this organometallic precursor is that the complete transformation to α-Al_2_O_3_ can be achieved in relatively lower temperature than reported in aluminum hydroxide based precursor. In this article, we present high density nucleation of α-Al_2_O_3_ nanocrystallites with less 10 nm in size from high purity aluminum oxide matrix by rapid heating of the aluminum formate hydroxide-based precursor powder. α-Al_2_O_3_ with hollow rod-like morphology finally formed through coalescence and growth of nanocrystallites after heating at 1200 °C for 1 min.

## Methods

Al(NO_3_)_3_ 9H_2_O (GR Nacalai tesque), ammonia solution (GR Nacalai tesque), and HCOOH (GR Nacalai tesque) were used as raw materials to prepare α-Al_2_O_3_. To prepare the precipitate of Al(OH)_3_, the pH of 0.5 M aluminum nitrate aqueous solution was adjusted to 6 by adding 1.5 M ammonia solution. The gel-like precipitate was separated from the supernatant solution by centrifugalization and washed by deionized water. Subsequently, HCOOH was added to the precipitate. The molar ratio of Al to HCOOH was 1 to 3. They were continuously stirred for 1 h and eventually turned into a transparent solution. The prepared transparent solution was dried in the oven at 150 °C for 24 h to prepare the precursor powder.

Pt crucible, which is 1 cm in diameter and 2 cm in height, was filled with 0.6 g of the precursor powder. The perpendicularly arranged tube furnace was heated to the temperature of 1200 °C in advance and then the Pt crucible hanged with Pt wire put into the furnace in 1 s. After the isothermal annealing for 10–100 s, the Pt crucible was removed from the furnace and quenched on the water cooled Cu plate. To prevent the reaction between Pt crucible and Cu plate, Al_2_O_2_ powder was placed on the Cu plate.

Phase identifications were performed by Rigaku MiniFlex600 X-ray powder diffraction (XRD) using CuKα radiation in the range of 2θ = 10–70° with a scanning speed of 2 °/min. The calcined powders were mixed with ethanol to make a suspension and subsequently a few droplets of it were used for microstructure evaluation by JEOL EM-2100 transmission electron microscopy (TEM). The change in the local structure of Al atoms during the transformation to α-Al_2_O_3_ was evaluated by ^27^Al MAS NMR technique. ^27^Al MAS NMR spectra were recorded by Bruker AVANCE III 500 spectrometer at 130.318 MHz with 15 kHz spinning speed, 4.0 μs pulses and 1 s relaxation time for 1,000 scans. 1.0 M AlCl_3_ aqueous solution was used as a chemical shift reference (0.1 ppm).

## Results

**Figure 1:**
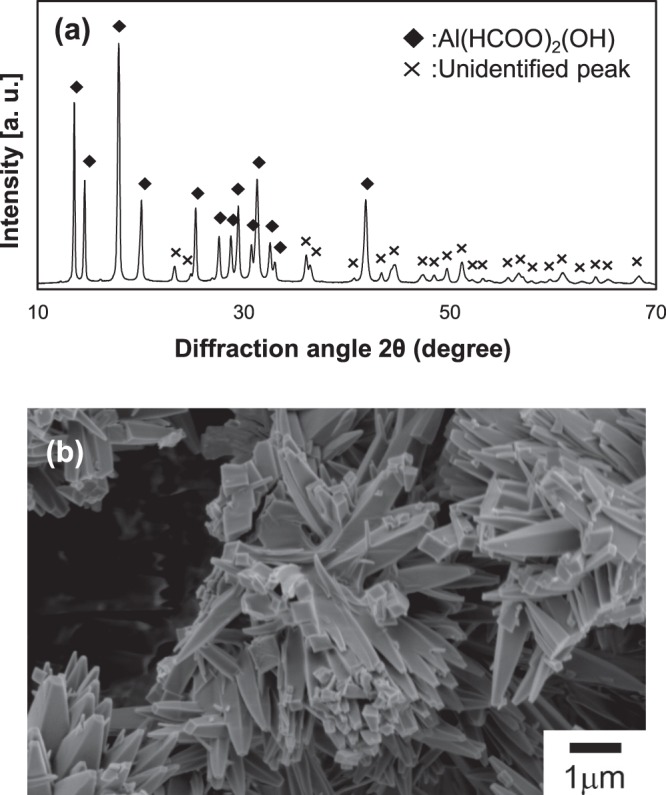
(**a**) XRD pattern using CuKα radiation and (**b**) SEM micrograph of the precursor powder.

Figure 1(a) shows XRD spectra of the precursor powder. Except for the peak at 2θ = 36° and some minor peaks in 2θ < 44°, the main crystal phase of the precursor powder was identified as aluminum formate hydroxide (Al(HCOO)_2_(OH)). Figure 1(b) shows the SEM micrograph of the precursor powder. The grains with rod-like morphology aggregated to make a flower-like structure.

**Figure 2:**
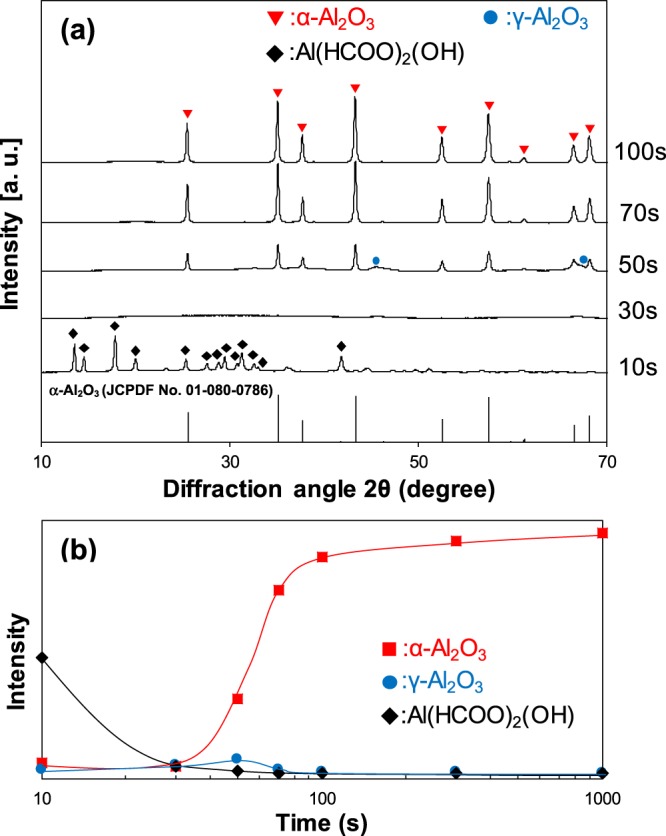
Evolution of the crystal phases during transformation to α-Al_2_O_3_ at 1200 °C; (**a**) a series of XRD spectra; (**b**) the peak intensities of α-Al_2_O_3_ (2θ = 43.3°), γ-Al_2_O_3_ (2θ = 67.6°), and Al(HCOO)_2_(OH) (2θ = 17.8°) for the samples heated for different soaking time. For comparison, the peaks of α-Al_2_O_3_ (JCPDF No. 01-080-0786) are also shown in the figure.

Figure 2(a) shows a series of XRD spectra for the samples during transformation to α-Al_2_O_3_ at 1200 °C. For comparison, the peaks of α-Al_2_O_3_ (JCPDF No. 01-080-0786) are also shown in Fig. 2(a). The peak intensities of α-Al_2_O_3_ (2θ = 43.3°), γ-Al_2_O_3_ (2θ = 67.6°), and Al(HCOO)_2_(OH) (2θ = 17.8°) for the samples heated for different soaking time are shown in Fig. 2(b). The samples maintained a powder state (not sintered) after an isothermal annealing for 10–100 s. When the sample was heated for 10 s, the peaks corresponding to Al(HCOO)_2_(OH) were detected. With increasing the soaking time to 30 s, no peaks were detected, indicating that aluminum formate was completely converted to amorphous alumina. With further increasing the soaking time to 50 s, the peaks corresponding to α-Al_2_O_3_ appeared, and the quite weak peaks corresponding to γ-Al_2_O_3_ were also detected. Subsequently, the peaks of γ-Al_2_O_3_ disappeared, and single phase of α-Al_2_O_3_ was obtained over 70 s duration. The crystallite size of α-Al_2_O_3_ estimated by Scherrer equation from (113) reflection varies from 38.7 to 42.7 to 40.9 nm on increasing the soaking time from 50 s to 70 s to 100 s (Supplementary data 1). The crystal densities of α-Al_2_O_3_ obtained from XRD for 50 s, 70 s and 100 s exhibited 3.99, 3.98 and 3.97 g/cm^3^, respectively. The crystal densities in the respective samples were higher than those of the transient phases (γ: 3.40 g/cm^3^ and θ: 3.58 g/cm^3^) and comparable to the reported value of α-Al_2_O_3_ (3.98 g/cm^3^)^6,8^ (Supplementary data 2).

**Figure 3:**
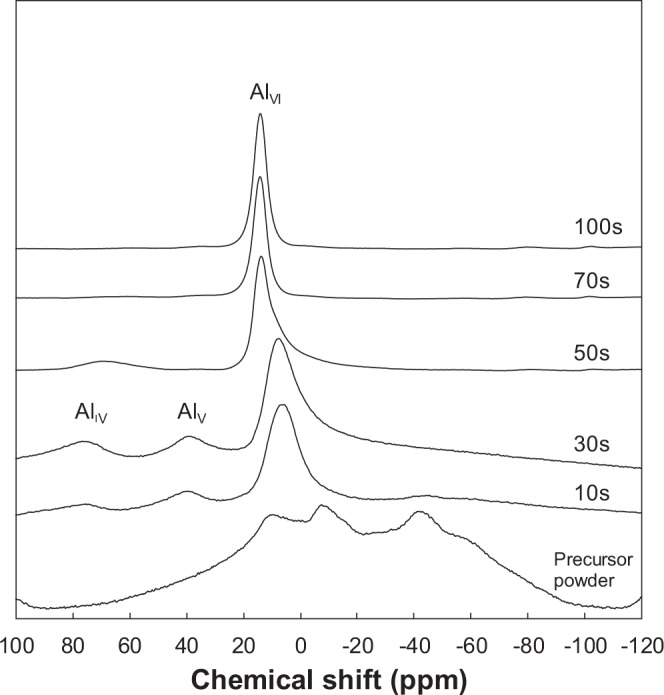
A series of ^27^Al MAS NMR spectra for the precursor powder calcined at 1200 °C. For comparison, the result for the precursor powder is also shown in the figure.

Figure 3 shows ^27^Al MAS NMR spectra for the precursor powders calcined at 1200 °C with various soaking time. For comparison, the result for the precursor powder is also shown in Fig. 3. The broad bands of the precursor powder observed at 9.91, −7.06 and −41.82 ppm could be attributed to octahedral aluminum species (Al_VI_). After calcination for 10 s, the bands at −7.06 and −41.82 ppm decreased drastically and new bands were observed at 38.79 and 74.54 ppm which were attributed to pentavalent (Al_V_) and tetrahedral (Al_IV_) aluminum species, respectively^17^. The bands at 9.91 ppm could be derived from the bidentated bond between aluminum and carboxyl group, while the peaks at −7.06 and −41.82 ppm could be associated with hydrogen bonds between water and aluminum formate^17^. From the observation of the band attributed to pentavalent aluminum species (Al_V_) not present in crystalline phases^18^, a part of aluminum formate was converted to amorphous alumina by calcination for only 10 s. With increasing the soaking time to 30 s, the bands at −7.06 and −41.82 ppm completely disappeared. The observed bands at 7.67, 39.29 and 74.39 ppm suggest that three types of the chemical state of aluminum species are in the amorphous alumina. With increasing the soaking time to 50 s, the spectra showed the typical signal of Al_VI_ in α-Al_2_O_3,_ and the band attributed to Al_V_ disappeared. The shoulder peak at 8 ppm produced by Al_VI_ in γ-Al_2_O_3_, and the second peak at 69.38 ppm corresponding to Al_IV_ in γ-Al_2_O_3_ were also observed in the sample soaked for 50 s^13^. This is consistent with the XRD result (Fig. 2(a)). With further increasing the soaking time, the bands attributed to Al_VI_ and Al_IV_ in γ-Al_2_O_3_ disappeared and finally, a single band attributed to the octahedral aluminum species in α-Al_2_O_3_ was detected at 14.07 ppm^19^. These results are in consistence with the previous report of ^27^Al MAS NMR spectra for aluminum formate calcined at 200–1500 °C^13^.

**Figure 4:**
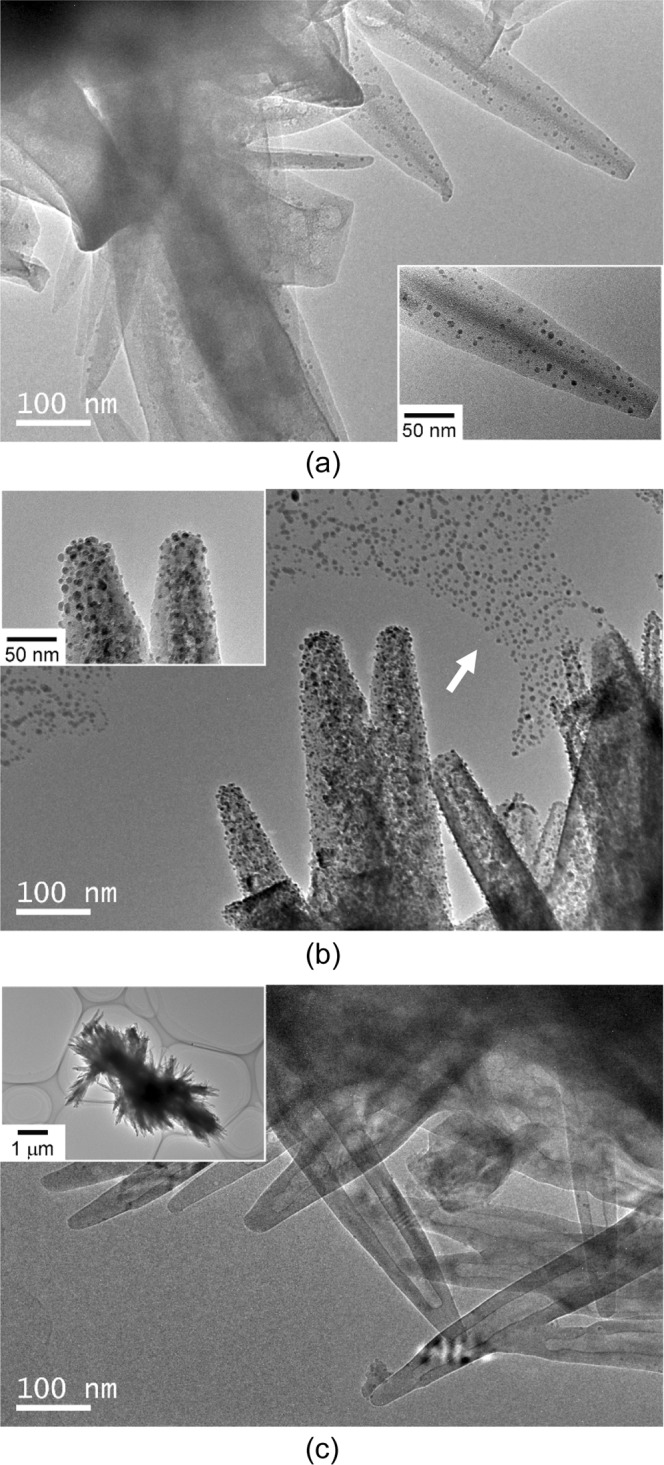
TEM micrographs of the samples heated at 1200 °C for 30 s (**a**), 50 s (**b**) and 100 s (**c**). The inset right below in (**a**) is the high magnification image showing the sparse nucleation. The inset left above in (**b**) highlights the high density nucleation of α-Al_2_O_3_ nanocrystallites. The inset left above in (**c**) is the low magnification image indicating the morphology of the aggregate.

Figure 4 shows TEM micrographs of the samples heated for 30 s, 50 s and 100 s, and then quenched from 1200 °C. The rod-like grains originated from the precursor powder were observed in the sample heated for 30 s as shown in Fig. 4(a). The high magnification image for 30 s showed the sparse nucleation of nanocrystallites in the rod-like grain. When the sample was heated for 50 s, the higher density of nucleation of nanocrystallites was observed in the rod-like grains. Moreover, a sea of nanocrystallites were also observed around the rod-like grains (indicated with white arrow in Fig. 4(b)). This might be the artifact during the preparation of TEM sample. These nanocrystallites observed on the collodion film are supposed to drop out of rod-like grains in the ethanol suspension. In the sample heated for 100 s, nanocrystallites were not observed and the hollow tubular structure was observed in the rod-like grains.

## Discussion

**Figure 5:**
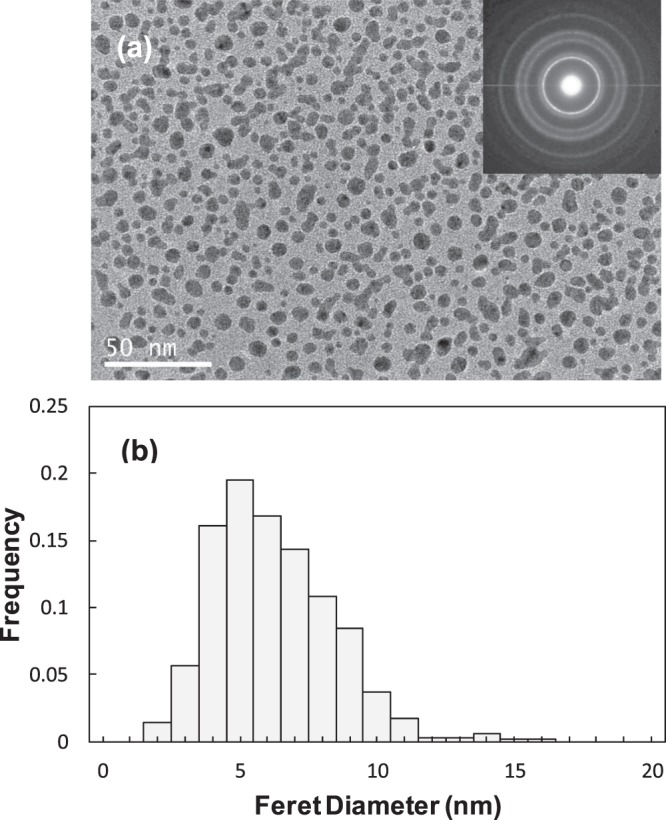
(**a**) TEM image and (**b**) size distribution histogram of the α-Al_2_O_3_ nanocrystallites.

Figure 5(a) shows the high magnification TEM image around the rod-like grains in Fig. 4(b). The TEM image of the nanocrystallites obtained at 1200 °C for 50 s revealed that the nanocrystallites are disperse and almost equiaxed in shape. The SAED analysis of the nanocrystallites in Fig. 5(a) shows a typical diffraction pattern of α-Al_2_O_3_. The size distribution histogram of the α-Al_2_O_3_ nanocrystallites [Fig. 5(b)] was determined by image analysis of the TEM images taken from about 700 particles. It revealed that the α-Al_2_O_3_ nanocrystallites had a mean size of 5.7 nm and a size distribution from 1.6 to 15.4 nm. The size distribution width of the α-Al_2_O_3_ nanocrystallites was narrow. The vermicular microstructure reported in literature was sometimes observed in our samples, but the size of the structure was not more than 10 nm, which was about one tenth of that reported in the literature^12^. These results suggested that the critical particle size for α-Al_2_O_3_ was ~10 nm in this study.

**Figure 6:**
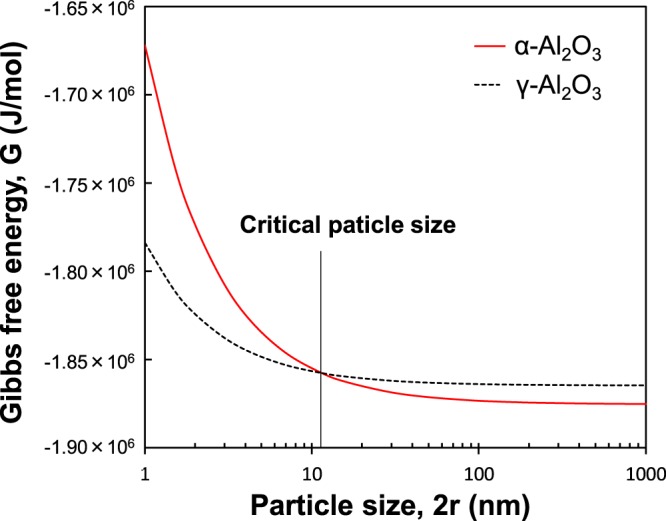
The relationship between Gibbs free energy, G and particle size, 2r at 1200 °C.

It was previously reported that γ to α phase transformation occurs by nucleation and growth processes in the γ-Al_2_O_3_ matrix. The critical particle size for the γ to α transformation depends on the surface free energy of the alumina phases. Increases in the Gibbs free energy of the alumina particles resulting from their small radii of curvature can be expressed as^20^:

1 $$G={G}^{0}+2\gamma {V}^{0}/r$$

where *G* is the Gibbs free energy of an alumina particle with radii of curvature *r*, γ is surface energy, *G*° is the standard Gibbs free energy, and *V*^0^ is the molecular volume. According to the literature^21,22^, the surface energy for α-Al_2_O_3_ (1.98 kJ/mol) was significantly higher than those of γ-Al_2_O_3_ (0.79 kJ/mol). Therefore, preferential exposure of the surfaces with lowest energy, γ-Al_2_O_3_ should become the energetically stable polymorph in the particle size below 11 nm at 1200 °C as shown in Fig. 6. This value corresponds approximately to the reported critical diameter of α-Al_2_O_3_^22^. According to the TEM image (Fig. 5(a)), the critical diameter of α-Al_2_O_3_ was much smaller than that reported in the literatures^23^.

The crystal structure of γ-Al_2_O_3_ is based on face-centered cubic packing of Oxygen (fcc), while the packing of Oxygen for α-Al_2_O_3_ is based on hexagonal close packing structure (hcp). It has been proposed that the activation energy for the γ to α transformation is related primarily to the rearrangement of oxygen sublattice^6^. This difference in the crystal structure between γ-Al_2_O_3_ and α-Al_2_O_3_ results in a disadvantage of γ-Al_2_O_3_ as the intermediate phase transforming to α-Al_2_O_3_. As clearly seen in the XRD result of Fig. 2(a) and the TEM micrograph of Fig. 4(a), α-Al_2_O_3_ nanocrystallites nucleated from the amorphous phase. In ^27^Al MAS NMR spectrum of the amorphous phase, the strong band and the broad bands attributed to octahedral (Al_VI_), pentavalent (Al_V_) and tetrahedral (Al_IV_) aluminum species were observed. It is well known that the local structure of aluminum atoms in α-Al_2_O_3_ is octahedral, while γ-Al_2_O_3_ is composed of both octahedral and tetrahedral coordinated aluminum atoms. The amorphous phase has no long range order, and the local structure of aluminum atoms in the amorphous phase is different from that in the crystalline phases. Nevertheless, the amorphous phase can easily transform to α- Al_2_O_3_ phase in a quite short calcination time. It has been reported that α-Al_2_O_3_ nanocrystallites with a size under 10 nm nucleate from α-Fe_2_O_3_ matrix, which has the same crystal structure with α-Al_2_O_3_^14^. This indicates that α-Fe_2_O_3_ matrix with the corundum structure can act to reduce the nucleation barrier and facilitate α-Al_2_O_3_ formation. These results suggested that the intermediate phase of the amorphous which formed through the thermal decomposition of the aluminum formate hydroxide had lower activation barrier to form α-Al_2_O_3_ phase than that of the transition phases, and resulted in the nucleation of α-Al_2_O_3_ with the size under 10 nm. On the other hand, the crystallite size of α-Al_2_O_3_ obtained by Scherrer equation in Fig. 2(a) for 50 s was 38.7 nm which was larger than the estimated critical diameter of 11 nm in the nucleation from the transition phase in Fig. 6. Considering the estimation of crystallite size in XRD, it is not sure whether the amorphous phase has the lower activation barrier to form α-Al_2_O_3_ than the transition phases. We do not have the valid explanation of this discrepancy between TEM micrograph and XRD in crystallite size. The careful discussion is needed to clarify the critical particle size of α-Al_2_O_3_ during the rapid heating of this precursor powder.

**Figure 7:**
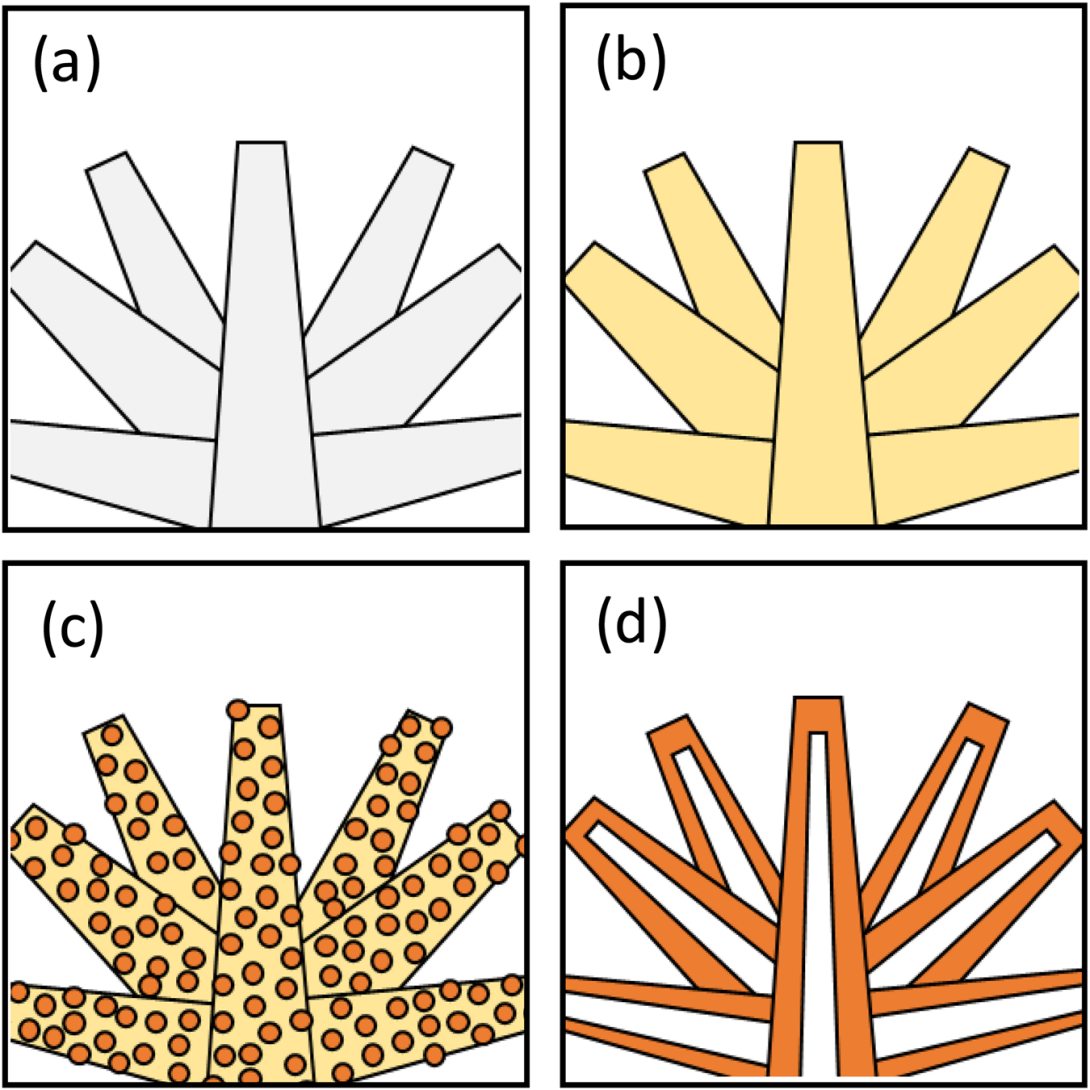
The microstructural evolution of the sample during the transformation to α-Al_2_O_3_; (**a**) the agglomeration of the rod-like aluminium formate hydroxide particles (Fig. 1(b)), (**b**) the precursor powder was thermally decomposed to form the amorphous phase (Fig. 4(a)), (**c**) the highly condensed nucleation of nanocrystallites in the rod-like grains (Fig. 4(b)) and (**d**) α-Al_2_O_3_ grains with hollow rod-like structure (Fig. 4(c)).

Figure 7 shows the schematic images of the microstructural evolution during the formation of the rod-like α-Al_2_O_3_. The agglomerations of the aluminum formate hydroxide particles were thermally decomposed to form the amorphous phase (Figs 1(b), 4(a)). Subsequently, the highly condensed nucleation of nanocrystallites was observed in the rod-like grains (Fig. 4(b)). Finally, the hollow tubular structure was observed in the grains (Fig. 4(c)). The observed hollow tubular structure suggests that α-Al_2_O_3_ nanocrystallites preferentially nucleate at the surface of the grains. α-Al_2_O_3_ powder is usually prepared by heat treatment of aluminum hydroxide such as Boehmite which strongly differ from each other in the morphology of the particles, depending on the conditions of synthesis. In the case of Boehmite, a calcination above 1200 °C for several hours is necessary for the complete transformation to α-Al_2_O_3_ due to the higher nucleation barrier^24^. Higher transformation temperature resulted in the activation of the mass transport which resulted in the deterioration of the controlled morphology of the particles. In our precursor powder, single phase of α-Al_2_O_3_ was successfully obtained in a quite short calcination time (about 1 min). The rod-like morphology of the precursor powder was maintained after a calcination achieving the complete transformation to α-Al_2_O_3_. The rod-like hollow α-Al_2_O_3_ is relatively lightweight and supposed to be easy to create three-dimensional network in the resin. Therefore, the rod-like hollow α-Al_2_O_3_ is expected to be a candidate material for a highly thermal conductive filler in power device.

In our previous report^16^, the complete transformation to α-Al_2_O_3_ successfully achieved by the normal furnace heating (5 °C/min) of the precursor powder consisting of aluminum formate hydroxide at 950 °C for 24 h, which is about 200 °C lower than that reported in the typical precursor of aluminum hydroxide. During the transformation to α-Al_2_O_3_ at 950 °C, nanocrystallites were not observed in the sample by TEM. Although the high density nucleation of α-Al_2_O_3_ nanocrystallites was observed in the sample heated at 1200 °C for 50 s, the resulting α-Al_2_O_3_ was not 100% phase pure. The residual amorphous phase and γ-Al_2_O_3_ persists. The results obtained in this paper indicates a possible beneficial effect of the rapid heating and cooling on the precipitation of α-Al_2_O_3_ nanocrystallites. As well as a raw material for the advanced nanoceramics, α-Al_2_O_3_ nano-particles are expected to be a candidate material for reducing environmental load such as catalyst support, gas separation membrane module and a fillar for transparent resin which facilitates the development of lightweight vehicle. We believe the combination of this precursor powder and the common rapid heating techniques such as IR, laser and gas burner would popularize a quantity synthesis of α-Al_2_O_3_ nano particles. To obtain 100% phase pure α-Al_2_O_3_ nano particles, it is necessary to optimize the synthesis conditions such as calcination temperature and heating rate.

## Conclusions

Homogeneous nucleation of α-Al_2_O_3_ nanocrystallites with less 10 nm in size through rapid heating of the aluminum hydroxide-based precursor powder was observed by TEM. α-Al_2_O_3_ nanocrystallites nucleated from the amorphous phase which formed after thermal decomposition of the precursor powder. ^27^Al MAS NMR spectra for the amorphous phase showed the strong band and the broad bands attributed to octahedral (Al_VI_), pentavalent (Al_V_) and tetrahedral (Al_IV_) aluminum species. These results suggested that the local structure of aluminum atoms in the intermediate phase of the amorphous was different from that in the crystalline phases. The crystallite size of α-Al_2_O_3_ observed by TEM in the sample heated for 50 s at 1200 °C was smaller value than the estimated critical diameter of 11 nm in the nucleation from the transition phase according to thermodynamics. On the other hand, the crystallite size of α-Al_2_O_3_ obtained by Scherrer equation exhibited 38.7 nm in the sample heated for 50 s at 1200 °C. There was a discrepancy between TEM micrograph and XRD in crystal size. Single phase of α-Al_2_O_3_ was successfully obtained in a quite short calcination time (about 1 min). The rod-like morphology of the precursor powder was maintained after a calcination achieving the complete transformation to α-Al_2_O_3_. The results obtained in this paper indicates a possible beneficial effect of the rapid heating and cooling on the precipitation of α-Al_2_O_3_ nanocrystallites. The combination of this precursor powder and the common rapid heating techniques would popularize a quantity synthesis of α-Al_2_O_3_ nano particles.

## Supplementary information

Supplementary figures
