# Supplementary material for: Homogeneous nucleation of corundum nanocrystallites by rapid heating of aluminum formate hydroxide-based precursor powder
Source: Sci Rep. 2019 Oct 17;9:14889. doi: 10.1038/s41598-019-51156-2 (PMC6797794; doi:10.1038/s41598-019-51156-2)

Supplementary data 1

Fig. X-ray diffraction in (113) reflection in α-Al_2_O_3_ in Fig. 2(a) at an expanded 2θ-scale.

Supplementary data 2

Table Crystal structure, lattice parameters, and lattice volume in the rapid heated samples

| Heat treatment | Crystallite size (nm) | Lattice parameters (nm) | | Lattice volume (nm^3^) |
| --- | --- | --- | --- | --- |
|  |  | a | c |  |
| 1200℃, 50s  α-Al_2_O_3_ | 38.7 | 0.4762 | 1.2980 | 0.2549 |
| 1200℃, 70s  α-Al_2_O_3_ | 42.7 | 0.4759 | 1.3015 | 0.2553 |
| 1200℃, 100s  α-Al_2_O_3_ | 40.9 | 0.4762 | 1.3021 | 0.2557 |

Supplementary data 3
TEM micrographs showing the homogeneous nucleation


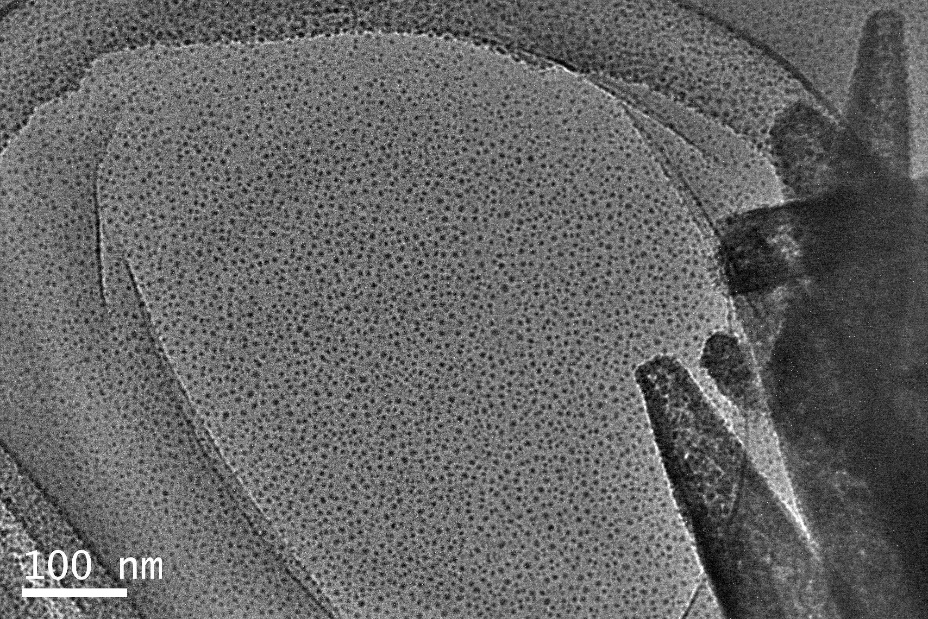


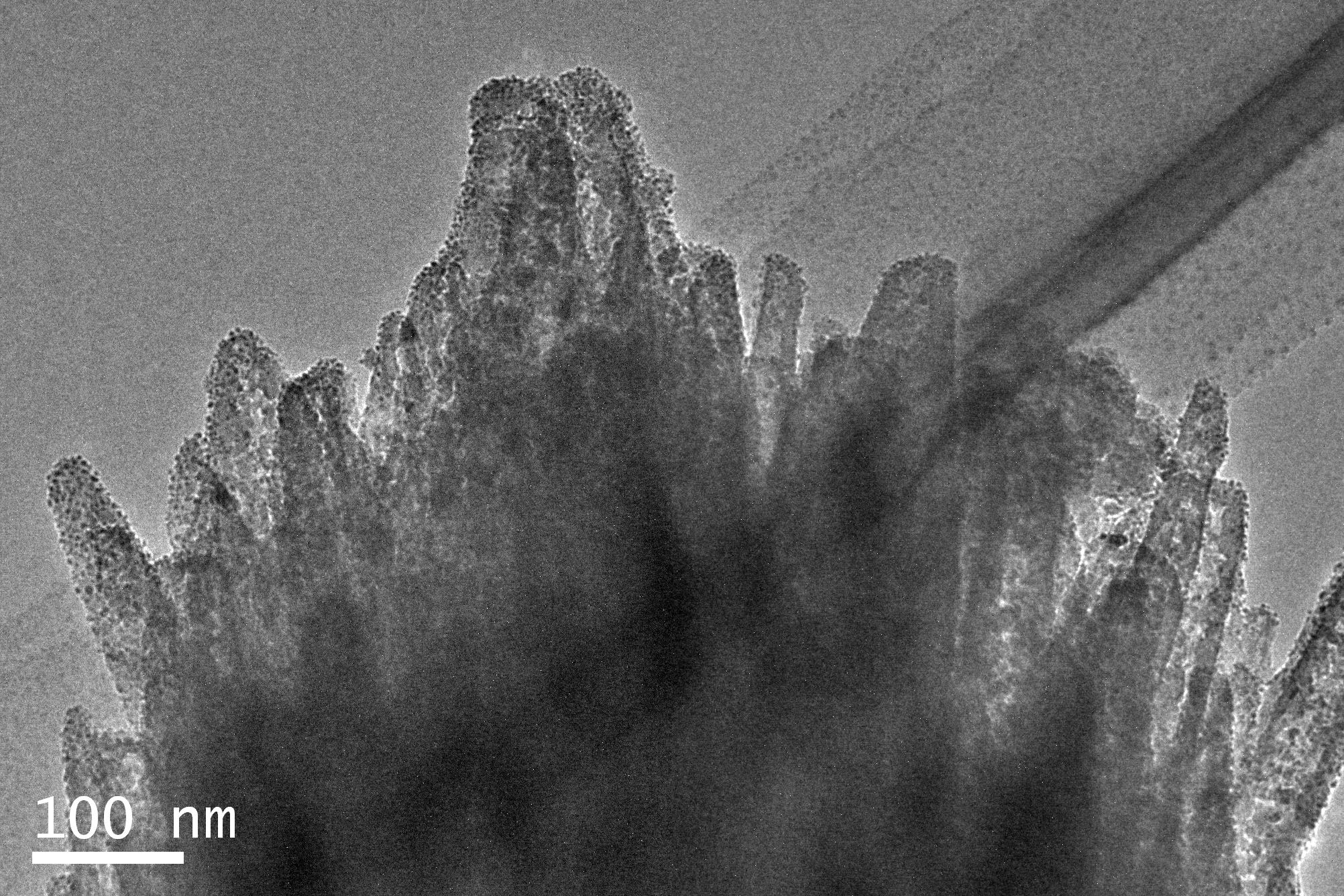

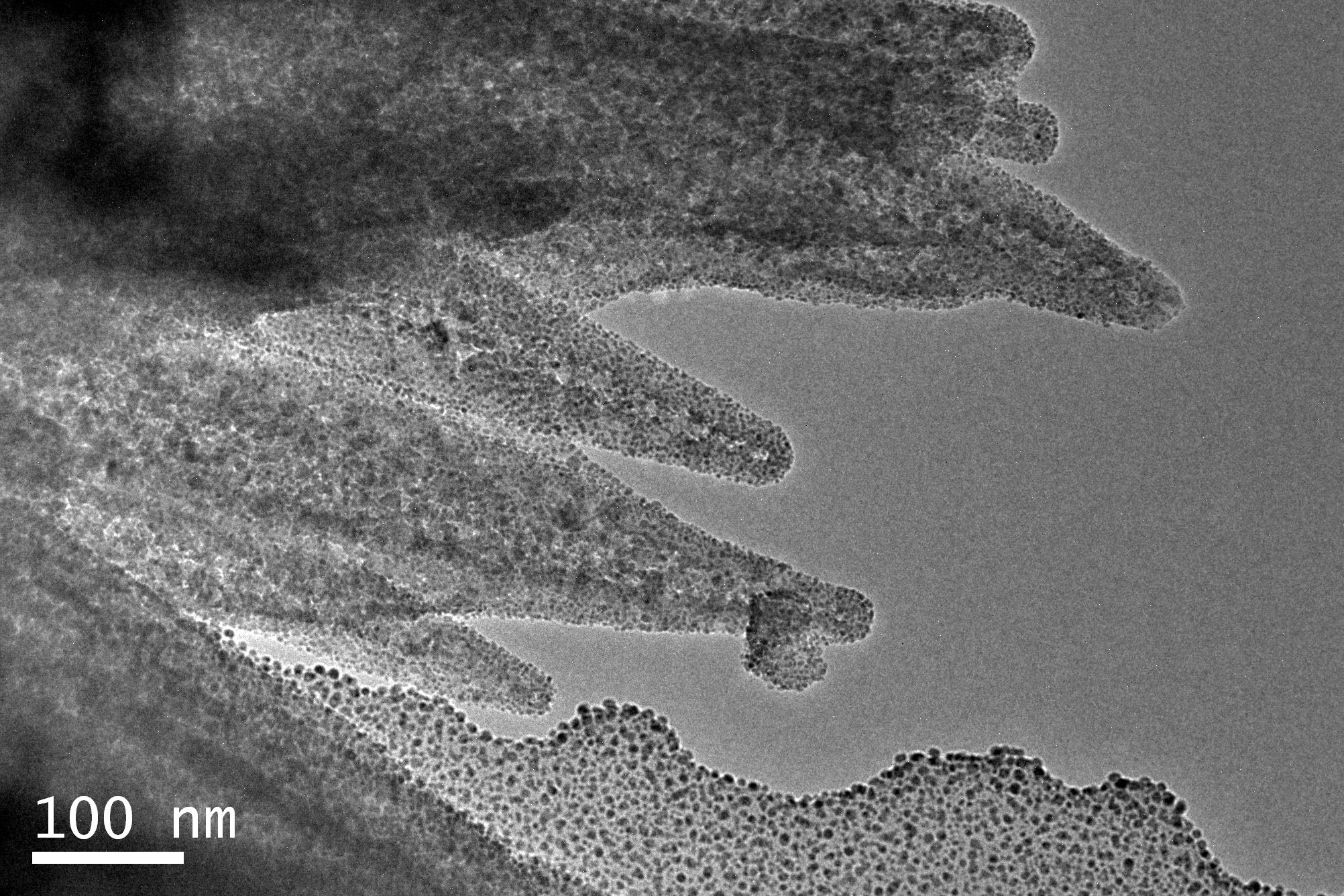


TEM micrographs showing the homogeneous nucleation


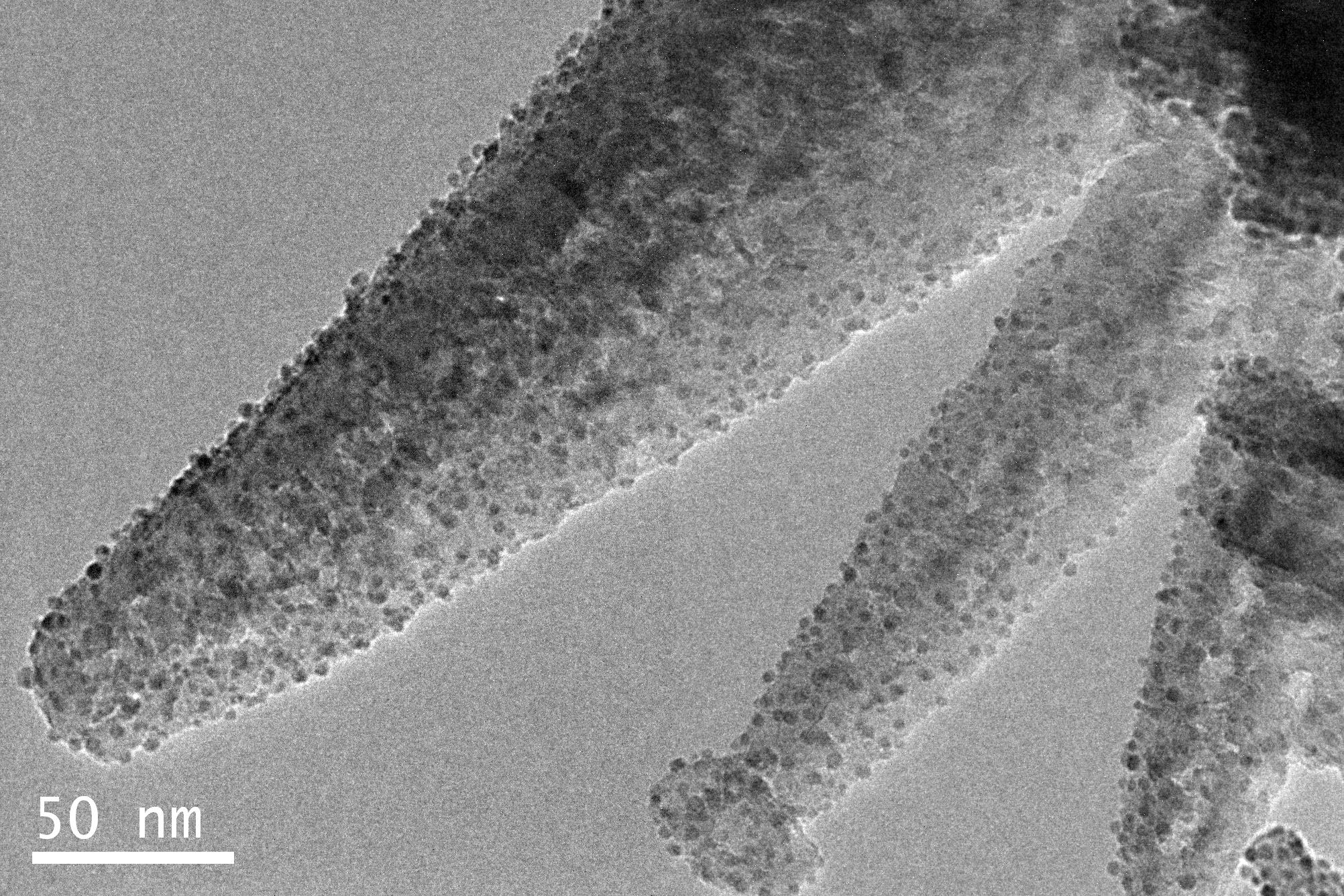

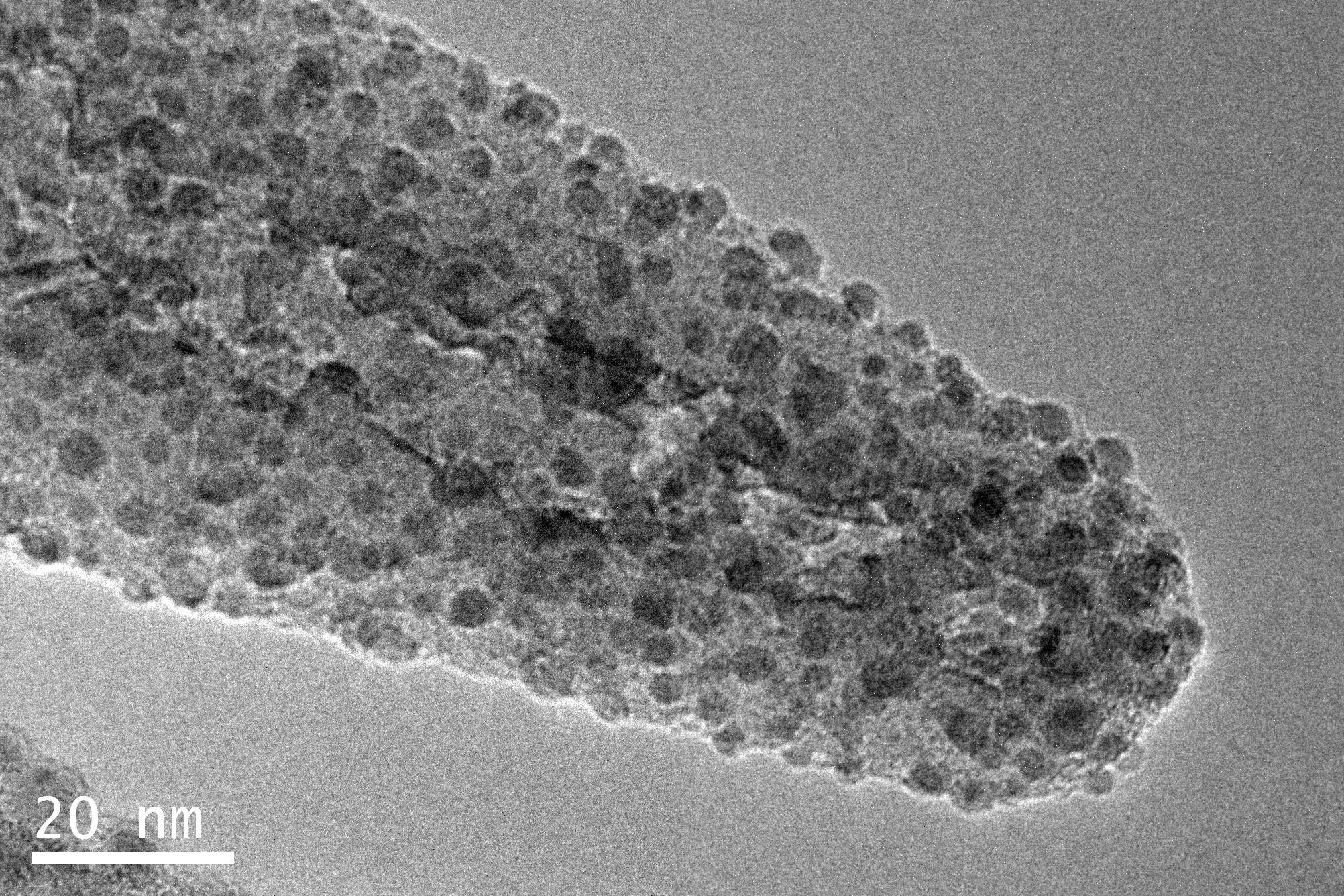

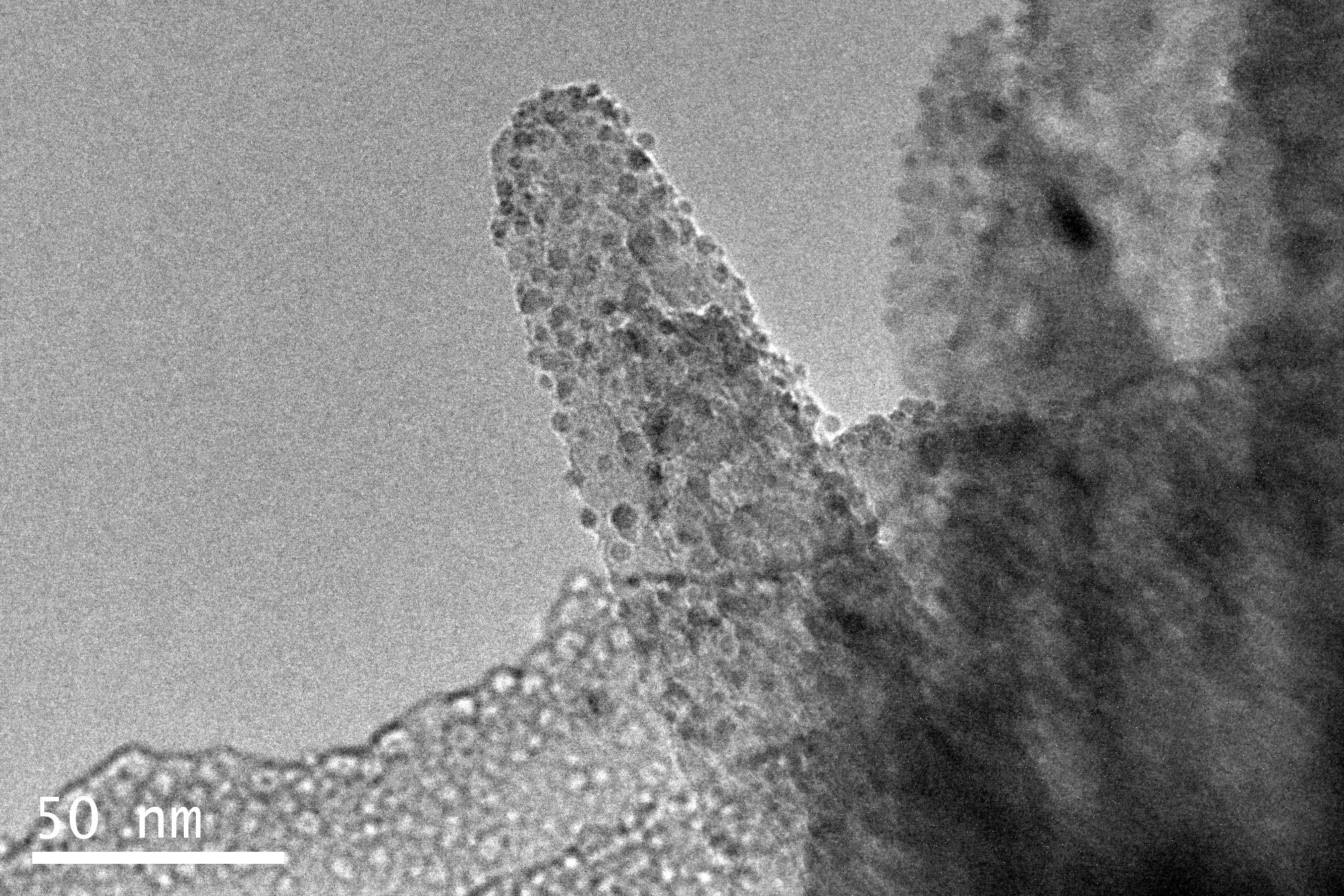

Supplement: Supplementary file 1 — Supplementary figures [file 41598_2019_51156_MOESM1_ESM.docx]
